# Long-Term Stability Improvement of Non-Toxic Dye-Sensitized Solar Cells via Poly(ethylene oxide) Gel Electrolytes for Future Textile-Based Solar Cells

**DOI:** 10.3390/polym12123035

**Published:** 2020-12-18

**Authors:** Jan Lukas Storck, Marius Dotter, Sonia Adabra, Michelle Surjawidjaja, Bennet Brockhagen, Timo Grothe

**Affiliations:** Faculty of Engineering and Mathematics, Bielefeld University of Applied Sciences, 33619 Bielefeld, Germany; marius.dotter@fh-bielefeld.de (M.D.); sonia.adabra@fh-bielefeld.de (S.A.); michelle.surjawidjaja@fh-bielefeld.de (M.S.); bennet.brockhagen@fh-bielefeld.de (B.B.); timo.grothe@fh-bielefeld.de (T.G.)

**Keywords:** dye-sensitized solar cells (DSSC), gel electrolyte, poly(ethylene oxide) (PEO), long-term stability, efficiency, dimethyl sulfoxide (DMSO), natural dyes, non-toxic

## Abstract

To overcome the long-term stability problems of dye-sensitized solar cells (DSSC) due to solvent evaporation and leakage, gelling the electrolyte with polymers is an appropriate option. Especially for future applications of textile-based DSSCs, which require cost-effective and environmentally friendly materials, such an improvement of the electrolyte is necessary. Therefore, the temporal progressions of efficiencies and fill factors of non-toxic glass-based DSSCs resulting from different gel electrolytes with poly(ethylene oxide) (PEO) are investigated over 52 days comparatively. Dimethyl sulfoxide (DMSO) proved to be a suitable non-toxic solvent for the proposed gel electrolyte without ionic liquids. A PEO concentration of 17.4 wt% resulted in an optimal compromise with a relatively high efficiency over the entire period. Lower concentrations resulted in higher efficiencies during the first days but in a poorer long-term stability, whereas a higher PEO concentration resulted in an overall lower efficiency. Solvent remaining in the gel electrolyte during application was found advantageous compared to previous solvent evaporation. In contrast to a commercial liquid electrolyte, the long-term stability regarding the efficiency was improved successfully with a similar fill factor and thus equal quality.

## 1. Introduction

To provide sufficient renewable energy in the future, solar cells are suitable for harvesting one of the main energy sources available to everyone [1]. Dye-sensitized solar cells (DSSCs) as third generation solar cells are a promising approach to utilize solar energy more environmentally friendly and cost-efficiently, because of the possibility to produce them from inexpensive, non-toxic materials without a cleanroom [2,3,4]. Since their discovery in 1991 [5] DSSCs are of commercial and academic interest and thus investigated intensively by a large number of research groups [6,7]. The best energy conversion efficiencies were achieved with rather toxic materials and liquid electrolytes, but those are associated with a problematic long-term stability due to leakage and sealing issues [7,8,9,10]. The electrolyte is commonly based on an iodine/triiodide redox couple and reduces the oxidized dye on the front electrode with an electron from the counter electrode [1,8]. Options to avoid leakage and evaporation of the volatile liquid electrolyte and to improve the long-term efficiency of DSSCs are polymer-based electrolytes among others like solid-state hole-transporting materials [8].

Polymer-based electrolytes, which can be produced by dissolving inorganic salts as redox couples without additional solvent in high molecular weight polymers, are advantageous in terms of mechanical properties and cost-efficiency [11]. For such solid-state polymer electrolytes, poly(ethylene oxide) (PEO), a non-toxic thermoplastic, is commonly used [8,12,13]. PEO is a semi-crystalline polymer with amorphous as well as crystalline phases at room temperature and can dissolve high concentrations of salts [14,15]. The ionic conductivity of this coordinating polymer is based on the segmental motion of polymer chains together with Lewis type acid-base interactions of the oxygen atom in the backbone of PEO and a cation, which makes both phases responsible for the ionic transport [14,16,17]. However, it is worth mentioning that the amorphous phase, in which the redox salts are primarily dissolved, is mainly responsible for ionic conductivity [15].

However, besides the advantages of minimizing leakage and solvent evaporation as well as improving long-term stability, solid-state polymer electrolytes also have the disadvantage of a relatively low ionic conductivity due to their relatively high crystallinity [11,17]. Combined with the comparatively poor contact to the dyed TiO_2_ layer, this results in an overall low device performance [11,17]. One way to overcome these issues is by complementing with additives, like plasticizers, to reduce the crystallinity of the polymer and to enhance therefore the ionic conductivity as well as the efficiency of DSSCs [14,17]. Alternatively, a solvent can be added to the polymer and electrolytic salts, which can also reduce the crystallinity and increase the polymeric chain mobility for better ionic conductivity, resulting in a quasi-solid-state or gel electrolyte [8,11,16]. In this case the polymer serves as a gelling agent of the liquid electrolyte and forms a three-dimensional matrix structure [8]. The formed gel electrolyte is in a quasi-solid-state as a hybrid system with transport characteristics of a liquid and cohesive properties of a solid [1,18]. In contrast to solid-state polymer electrolytes, gel electrolytes have advantages regarding contacting the counter electrode and the front electrode, due to a better penetration into the porous TiO_2_ layer [1,2,19]. Additionally, gel electrolytes have a low vapor pressure while maintaining an ionic conductivity close to that of liquid electrolytes [1,2,19].

Polymer-based gel electrolytes are of high interest regarding textile-based DSSCs, which have severe electrolyte evaporation problems because of a sealing inability due to the otherwise lost textile structure [10,20]. The challenge faced here is to gain a suitable gel electrolyte to improve fully textile DSSCs utilizing low cost, non-toxic and environmentally friendly materials so that the DSSCs can be properly integrated into clothing or large-scale textile architecture, which would be a severe advancement and overcome the problem of typically small energy conversion efficiencies in the future [10,21,22].

As a first step, this paper investigates the applicability of common PEO-based gel electrolytes without plasticizers and with focus on a high long-term stability for unsealed glass DSSCs with non-toxic materials. In this context PEO is well suitable due to the non-toxicity, low cost and reported good performances [23]. The commonly used solvent acetonitrile is not considered very suitable due to its toxicity, so distilled water and dimethyl sulfoxide (DMSO), which is labelled low toxic and environmentally friendly, are suggested as appropriate alternatives [8,24]. Furthermore, a natural anthocyanin-based dye is used as a low-cost and non-toxic alternative to conventional, expensive and toxic dyes, such as N3 or N719, to develop environmentally friendly DSSCs suitable for textile integration [10,25].

## 2. Materials and Methods

All DSSCs were assembled with fluorine-doped tin oxide (FTO) coated glasses (Man Solar, Petten, The Netherlands) as electrodes. The front electrodes were purchased with a pre-coated TiO_2_ layer and the counter electrodes were treated with a 6B graphite pencil (Faber-Castell, Stein, Germany) to gain a catalyst layer. Forest fruit tea (Mayfair, Wilken Tee GmbH, Fulda, Germany), which includes anthocyanins and was previously tested as one of the most efficient non-toxic and inexpensive dyes, was used to dye the TiO_2_ layer under mild conditions [26]. In this regard, the dye was extracted for 15 min at room temperature by stirring 10 g tea in 120 mL distilled water, which is reportedly a suitable solvent for dye extraction of anthocyanins [27]. Then filtration was conducted, before the TiO_2_ layered electrodes were inserted in the permeate for 10 min. Subsequently, the front electrodes were rinsed with distilled water and dried at ambient conditions.

The compositions of the investigated gel electrolytes are depicted in Table 1. The gel electrolytes were based on PEO with an average molecular mass of 600 kg/mol (S3 Chemicals, Bad Oeynhausen, Germany), which was dissolved in distilled water or DMSO (Carl Roth, Karlsruhe, Germany). Potassium iodide (KI) (VWR, Darmstadt, Germany) and molecular iodine (I_2_) (Carl Roth, Karlsruhe, Germany) in a weight ratio 2:1, as in Lugol’s solution, were used as inorganic salts for the iodine-triiodide redox couple. For each gel electrolyte the components were gradually added to the solvent at 70 °C and stirring was continued for at least 1 h. Different amounts of DMSO, which affected the concentration of electrolytic salts and PEO, had to be added to different samples in order to obtain a similar consistency with adequate coatability. After dissolving of the components each warm gel electrolyte was coated by a box-type doctor blade with a defined wet layer thickness of 30 µm on three graphite coated counter electrodes.

Optional solvent evaporation of the gel electrolyte, which for the sake of convenience is here referred to as drying, was carried out at ambient conditions for different time periods or with a laboratory oven (Memmert, Schwabach, Germany). DSSCs were then assembled by placing the front electrode on the gel electrolyte layer of the counter electrode and fixed with transparent tape without additional pressure, resulting in an active energy conversion area of 6 cm^2^ each. Three DSSCs with commercial fluidic iodine-triiodide electrolyte (Man Solar) were built as a reference. Here the electrolyte was filled in through capillary action after assembly of the electrodes. Again, no external pressure was applied, so that complete pore filling, together with an efficiency increase, required about three days, as experienced [21,28,29]. Initially, three DSSCs per sample were assembled and in the experimental progress, the best DSSC was selected and is presented here.

In order to assess the progression of the photovoltaic performance resulting from efficiency and fill factor of the unsealed DSSCs, current-voltage (I-U) curves were measured at room temperature against a black background with a Keithley 2450 sourcemeter while DSSCs were illuminated with 100 mW/cm^2^ by an LS0500 solar simulator with AM 1.5 G spectrum (LOT-Quantum Design GmbH, Darmstadt, Germany). Between the measurements, DSSCs were stored in the dark at room temperature. Based on the measurements, the fill factors and efficiencies were calculated to plot the temporal progressions. All DSSCs were measured for 52 days in order to investigate the influence of the gel electrolyte composition and to determine the suitability of the gel electrolyte with regard to an age-related change in the cells.

Additionally, the absorption spectrum of the forest fruit tea was recorded with a Thermo Scientific Genesys 10S UV/Vis spectrophotometer (Fisher Scientific, Schwerte, Germany) at a 1:30 dilution of the dye extract solution used for dyeing. An Axio Observer microscope (Carl Zeiss, Göttingen, Germany) was used to take reflected-light microscopic images of layers of a DSSC. The ionic conductivities of the investigated electrolyte solutions were measured at 40 °C with a LWT-01 Voltcraft conductivity test pen (Conrad Electronic, Wollerau, Switzerland).

## 3. Results and Discussion

**Figure 1:**
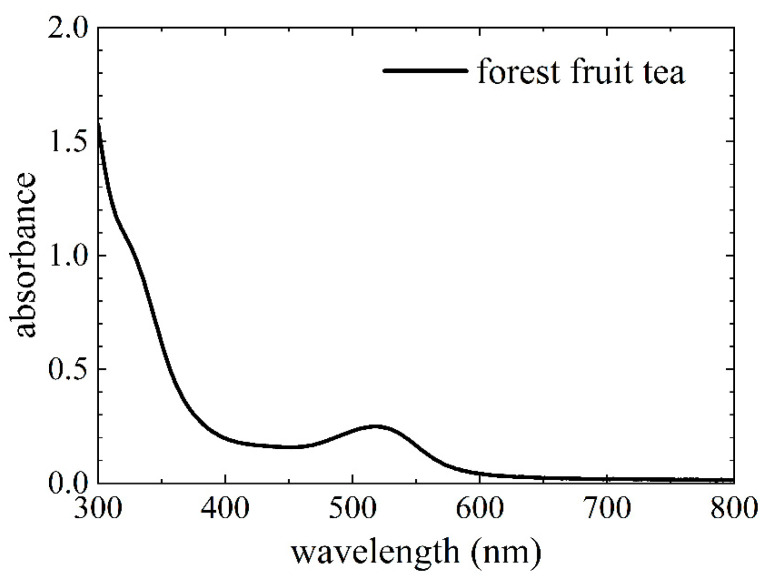
Absorption spectrum of the forest fruit tea dye.

At first, the dye extracted from the forest fruit tea was characterized. The absorption spectrum of the obtained dye is depicted in Figure 1.

The spectrum of the forest fruit tea has a broad peak within the visible range around 520 nm (cf. Figure 1), which shows the presence of anthocyanins [26]. Anthocyanins are considered to be well suited natural dyes for DSSCs [28]. Although the recipe of forest fruit tea has changed, the absorption spectrum is still very similar to the spectra of dyes extracted from forest fruit tea in previous publications [28,30,31].

Based on the composition of the gel electrolyte used in the work of Syairah et al. [2], preliminary tests were carried out due to the omission of ionic liquids and in order to reach a suitable composition for our proposed gel electrolyte. This resulted in the gel electrolyte compositions listed in Table 1. The ionic conductivities of the different gel electrolyte solutions and of the commercial liquid electrolyte of the reference were measured and are depicted in Table 2.

**Figure 2:**
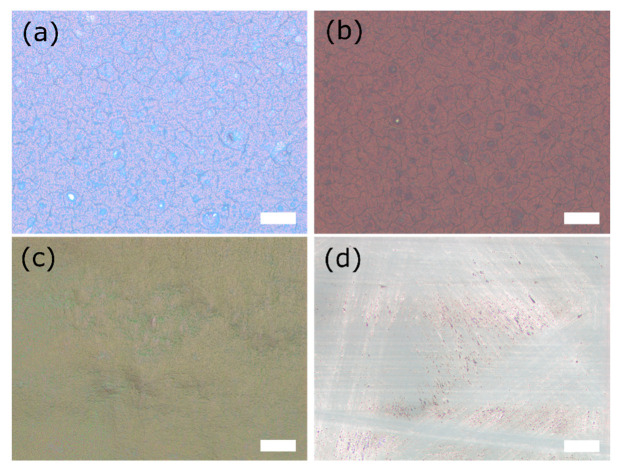
Reflected-light microscope images of DSSC components in 10× magnification: (**a**) TiO_2_ layer of the front electrode, pre-coated on a glass electrode by Man Solar; (**b**) With forest fruit tea dyed TiO_2_ layer of the front electrode; (**c**) Gel electrolyte of sample 1, 1.5 h after coating with a box-type doctor blade on a glass slide; (**d**) Counter electrode, coated with graphite pencil. Scale-bars indicate 100 µm.

Furthermore, microscope images of the functional layers of the DSSC are presented in Figure 2. The pre-coated TiO_2_ layer on a conductive glass slide of the front electrode is in Figure 2a depicted. Figure 2b shows the TiO_2_ layer after dyeing with the anthocyanins from the forest fruit tea. Additionally, the coated electrolyte layer of the gel electrolyte of sample 1 can be recognized as a homogeneous layer in Figure 2c. The other gel electrolyte layers are not shown because microscope observation did not reveal significant differences. In Figure 2d the graphite coated counter electrode is presented with noticeable lines from the graphite pencil.

I-U curves were recorded to assess the long-term stability depending on the gel electrolytes, since this measurement provides information about the long-term performance of the gel electrolytes via the calculated efficiency without affecting the functionality of the DSSCs. During the experiment it was noticed that the efficiencies of the three specimens per sample fluctuated strongly. From the second week onwards, individual DSSCs began to deteriorate until their functionality ceased. The timing of this failure seems to be random and not reproducible. This is probably due to the known problem of low reproducibility of DSSCs, which is mainly based on differing pressures applied to the electrodes and on the large amount of manual work necessary in the laboratory [10,30]. Since this study focuses on the comparison of gel electrolytes, opposite to another related study dealing with the problem of low reproducibility of DSSCs comprehensively [32], here the comparison of the gel electrolytes is based on the best DSSC per sample and thus on the efficiencies achievable in an optimal case.

### 3.1. Influence of DMSO and Distilled Water as Solvents on Photovoltaic Performance

Distilled water and DMSO can each dissolve PEO and are therefore compared as possible non-toxic and environmentally friendly solvents. Both solvents are utilized for gel electrolytes concerning DSSCs in scientific literature. On the one hand, water is e.g., used as a solvent for xanthan gum based gel electrolytes by Park et al. [33]; on the other hand, DMSO is e.g., used as solvent for gel electrolytes in combination with polyvinyl alcohol or along with polycarbonate as solvent for agarose and ionic liquid based gel electrolytes [34,35].

**Figure 3:**
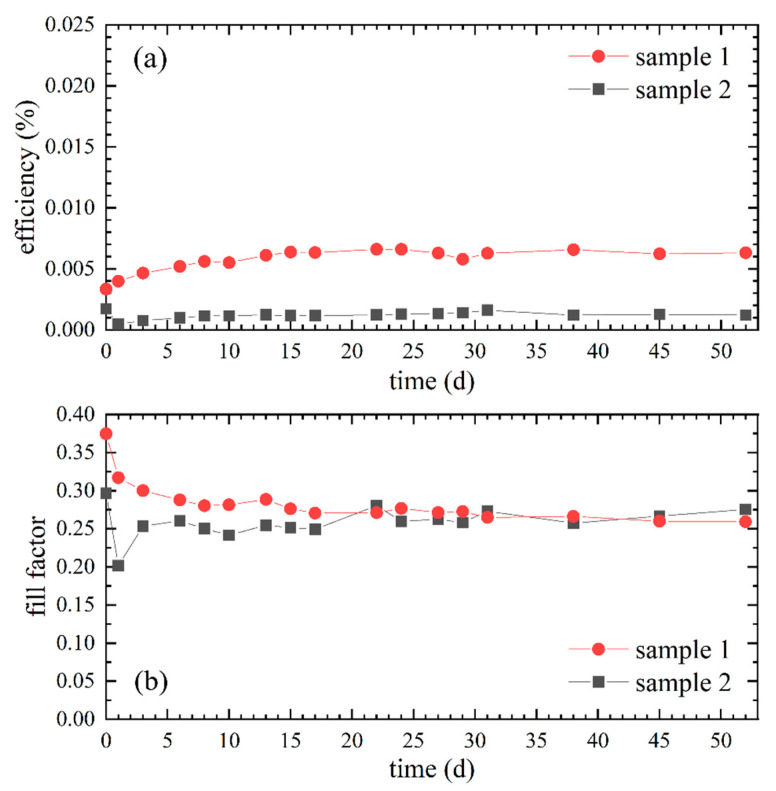
Efficiencies and fill factors of PEO gel electrolyte DSSCs assembled with DMSO and distilled water as solvents measured over 52 days: (**a**) Efficiencies of sample 1 with DMSO as solvent and of sample 2 with distilled water as solvent; (**b**) Corresponding fill factors of sample 1 and 2.

The ionic conductivity of the water-based gel electrolyte of sample 2 was (4.76 ± 0.01) mS/cm and thus almost twofold the ionic conductivity of the DMSO-based gel electrolyte of sample 1 with (2.85 ± 0.01) mS/cm (cf. Table 2). In contrast to the hypothesis that a higher ionic conductivity also results in a higher efficiency, the efficiencies of sample 1 were higher than those of sample 2 over the entire period, as depicted in Figure 3a. Regarding sample 1, a slight efficiency increase is observable in the first days, whereas the efficiency of sample 2 dropped during the first day. The fill factor (Figure 3b) of sample 1 decreased to values similar to the fill factor of sample 2, which fluctuated during the first days until it remained relatively constant. With a decreasing fill factor in sample 1, an increasing internal resistance can be assumed, indicating further aging.

A possible reason for the higher efficiency of the DSSC with DMSO as solvent compared to distilled water could be the lower vapor pressure of DMSO [36]. Consequently, DMSO is less likely to evaporate out of the gel electrolyte than distilled water and therefore the amorphous phase, in which most of the ionic conduction occurs, remains longer unchanged [15]. Evaporation of the solvent and drying of the gel electrolyte are in general associated with a decrease in ionic conductivity due to the increase of microscopic viscosity [34]. Drying of the electrolyte of the assembled DSSCs is generally possible because the DSSCs, like future textile-based ones, are unsealed and the solvent can evaporate.

Water is known to cause desorption of the dye molecules from the TiO_2_ by hydrolyzing the linkages between them and to influence the long-term stability of DSSCs negatively [33,37,38]. Additionally, a decrease in diffusion coefficient is related to water [33]. However, water appears to be a promising solvent for environmentally friendly DSSC electrolyte systems in some recent studies [8,39,40,41,42]. Hence, a water-based electrolyte (sample 2) was also investigated in this paper.

The mentioned negative properties of water regarding electrolytes for DSSCs are possible reasons for the overall low efficiency of sample 2 despite the high ionic conductivity of the water-based gel electrolyte. A further cause could be the comparatively faster drying of the water-based gel electrolyte. Based on these considerations and the data shown in Figure 3, DMSO is a more suitable solvent for our DSSCs and therefore used in the further experiments.

### 3.2. Influence of the PEO Concentration on Photovoltaic Performance

Samples 1 and 3 to 5 were built to investigate the influence of the PEO concentration in the gel electrolyte on the long-term DSSC photovoltaic performance. The ionic conductivities of the respective gel electrolytes of those samples are depicted in Table 2. Sample 4 with the highest PEO concentration (24 wt%) had with (2.71 ± 0.01) mS/cm the lowest ionic conductivity. With decreasing PEO concentration, ionic conductivity increases via (2.85 ± 0.01) mS/cm with 17.4 wt% PEO in sample 1, (3.15 ± 0.01) mS/cm with 9.5 wt% PEO (sample 3) to (3.21 ± 0.01) mS/cm with 8 wt% PEO (sample 5).

This trend corresponds to the results of Shi et al. [43], who also measured a decrease in ionic conductivity when the PEO concentration increased from 8 wt% to 15 wt%. Their gel electrolyte with a PEO concentration of 8 wt% had a ionic conductivity of about 8.75 mS/cm, which is approximately 2.7 times higher than the ionic conductivity of sample 5 with likewise 8 wt% PEO [43]. This difference is probably due to the usage of ionic liquids and PEO with a different molecular weight by Shi et al. [43]. The reason for lower ionic conductivities at higher polymer concentrations is considered to be an increase in viscosity and the reduction of free volume and amorphous phase, where most ionic conduction takes place, due to the more pronounced polymer matrix [7,15,43,44].

**Figure 4:**
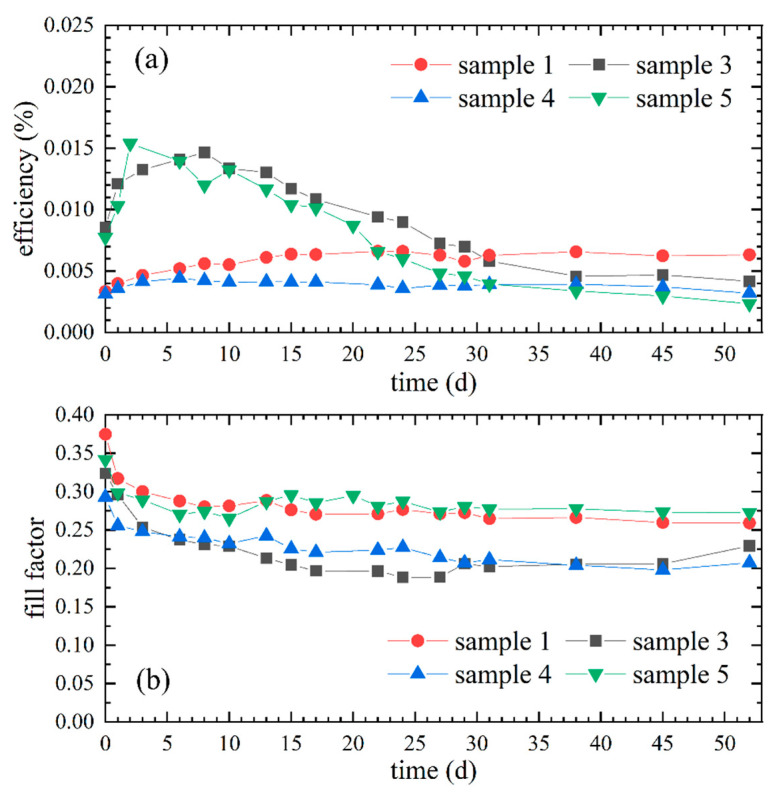
Comparison of samples 1 and 3 to 5 with regard to the potential influence of the PEO concentration in the gel electrolyte with 8 wt% in sample 5, 9.5 wt% in sample 3, 17.4 wt% in sample 1 and 24.0 wt% in sample 4: (**a**) Temporal progressions of the efficiencies of the corresponding samples; (**b**) Illustration of the respective fill factors over time.

The progressions of the efficiencies of the corresponding DSSCs are presented in Figure 4a and the fill factors are presented in Figure 4b. Remarkable are the increases in efficiency of samples 3 (9.5 wt% PEO) and 5 (8 wt% PEO) until day 10 and the subsequent constant decreases in efficiency. In contrast, the efficiencies of samples 1 (17.4 wt% PEO) and 4 (24.0 wt% PEO) increased moderately during the first days and remained relatively constant thereafter. After 30 days sample 1, whose efficiency was over the entire time period above that of sample 4, had the highest efficiency, due to the decreases of sample 3 and 5. Sample 1 and 5 show the highest fill factor values in the observed period and sample 3 and 4 the lowest, indicating a lower internal resistance of samples 1 and 5. However, a slight decrease of the fill factor can be observed in all samples.

According to the higher ionic conductivities, samples 3 and 5 exhibited higher efficiencies in the first three weeks (cf. Figure 4a) compared to samples 1 and 4, which had higher PEO concentrations. With advancing time and probable drying of the gel electrolyte, the content of DMSO decreases and the relative concentration of PEO increases, which could explain the observed subsequent decrease in efficiency of samples 3 and 5. In contrast, the efficiencies of samples 1 and 4 remained relatively constant after moderate increases, probably due to the initially higher PEO concentration, with which more stable DSSCs are associated [43].

However, despite the decreasing ionic conductivity in connection with increasing polymer concentration, Shi et al. [43] observed with an increasing PEO concentration an increasing stability of DSSCs and also an increase in efficiency up to a maximum PEO concentration of 10 wt%. With further increasing PEO concentration a decrease in photovoltaic performance was reported. Referring to the results shown in Figure 4, sample 3 had a PEO concentration of 9.5 wt% and had also a high efficiency in the first days. Higher PEO concentrations resulted in lower efficiencies too. Besides the connection between higher ionic conductivity and higher efficiency, the data in Figure 4a also show a connection between higher PEO concentration and higher stability of DSSCs. Thus, these results are comparable to those of Shi et al. despite the different gel electrolyte systems.

The PEO concentration of sample 1, namely 17.4 wt%, seems to be the most suitable for the here presented environmentally friendly DSSCs, because it results in the most stable DSSC while maintaining high efficiency. It can be assumed that this PEO concentration prevents drying of the gel electrolyte for at least 52 days due to a not observed efficiency decrease. In addition, the efficiency of sample 1 is higher than the efficiency of sample 4, which had a higher PEO concentration and was apparently also stable over 52 days.

### 3.3. Influence of Drying before Assembly on Photovoltaic Performance

In scientific literature, DSSCs are assembled on the one hand after drying of the gel electrolyte [16,44,45]. On the other hand, drying is not explicitly implemented and the solvent remains in the gel electrolyte as the DSSCs are assembled [2,15,19,35]. Sometimes it is not exactly characterized to what extent drying is carried out [43,46]. Here, gel electrolytes were dried under different conditions (cf. Table 1) to investigate the influence of a potential drying before DSSC assembly on the efficiencies and fill factors concerning their temporal progressions.

**Figure 5:**
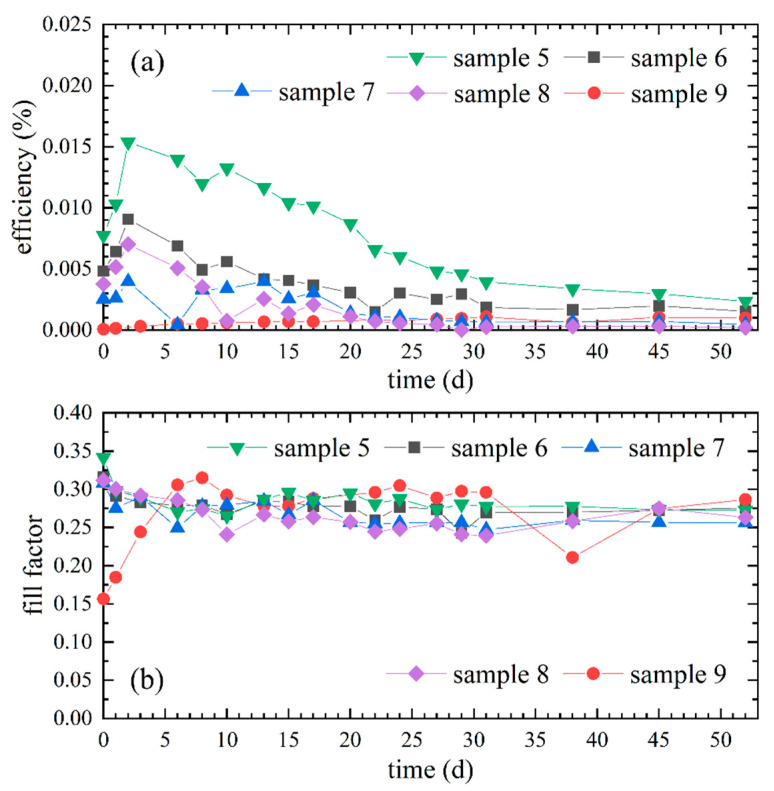
Influence of gel electrolyte drying before DSSC assembly on the progression of photovoltaic performance: (**a**) Efficiencies of sample 5 with a not dried gel electrolyte, sample 6, 7 and 8 with at room temperature dried gel electrolytes for 0.5 h, 1 h and 2 h, respectively, and sample 9 with a gel electrolyte dried in the oven; (**b**) Corresponding fill factors of these DSSCs with differently dried gel electrolytes.

In Figure 5a the resulting efficiencies of the differently dried samples 5 to 9 are depicted. Sample 5, for which the gel electrolyte was not dried, had the highest efficiency over the entire period in comparison to the samples with dried gel electrolytes. The efficiencies of samples 6 to 8, dried in a range of 0.5 h to 2 h, were relatively analogous and in their general progressions similar to sample 5, but with lower values. In contrast, the efficiency of sample 9 had a distinct different progression after drying in the oven, consisting of a slightly increasing efficiency from a very low value. These observations indicate a negative influence of the performed drying of the gel electrolyte prior to assembly on the efficiency.

The temporal progressions of the respective fill factors of samples 5 to 8 are relatively similar and shown in Figure 5b. During the first days a decrease is notable, which could indicate an increased internal resistance, possibly due to further drying of the gel electrolyte. Again, sample 9 is characterized by a considerable difference in progression due to an increase of the fill factor during the first 8 days.

Despite the relatively low vapor pressure of DMSO, drying for 0.5 h seems to evaporate enough solvent to have a negative influence on efficiency [36]. However, the efficiency of sample 6 does not differ as much from the efficiencies of samples 5, 7 and 8 as it does from the efficiency of sample 9. This is most probably due to the drying of the gel electrolyte of sample 9 in the oven, resulting in an explicitly enhanced amount of evaporated solvent.

In general, scientific literature commonly states that low energy conversion efficiencies and fill factors in DSSCs with gel electrolytes are caused by poor contact between the gel electrolyte and the dye due to the crystallinity and poor penetration of the electrolyte into the nanopores of the semiconductor layer on the front electrode [6,8,11,17,44]. With a more fluidic electrolyte, which can be achieved by a higher solvent content, a better penetration into the TiO_2_ pores is reached, which leads to more efficient dye regeneration and to better photovoltaic performance [11,16,44]. In this respect it is to mention that the gel electrolyte was coated on the counter electrode and then dried, thus the penetration of the gel electrolyte into the dyed semiconductor layer started after DSSC assembly. Consequently, this penetration for sample 9 was probably hindered by the high viscosity and low solvent content of the respective gel electrolyte at the assembly time due to previous drying in the oven.

Additionally, the gel electrolyte of sample 9 was prepared with a higher PEO concentration than samples 5 to 8, which further increased the viscosity and crystallinity [7,15,43]. Resulting from this, it is hypothesized that the gel electrolyte of sample 9, in addition to hindering the penetration into the TiO_2_ pores, also had a reduced portion of the amorphous phase with less free volume, which led to lower ionic conductivity and inferior photovoltaic performance [7,15,43].

The worse penetration into the semiconductor layer by the gel electrolyte of sample 9 due to a comparatively low solvent content is not only a possible reason for the low efficiency but is also part of an explanation for the increasing efficiency and fill factor over time. Usually, especially without additional pressure on the electrodes, it takes some time until the infiltration of the electrolyte through the pores of the TiO_2_ layer is completed, which is why an increase in efficiency is often observed in the first days (cf. samples 3 and 5 in Figure 4 as well as samples 6 and 8 in Figure 5) [21,28,29,47]. Apparently, this time span is increased regarding sample 9 due to the more extensive, previous drying and resulting higher viscosity, which hinders and delays the diffusion into the TiO_2_ pores. In this context, Sonai et al. [47] stated likewise that the infiltration of a more viscous gel electrolyte into the TiO_2_ pores is slower than with a less viscous electrolyte.

These considerations and the results depicted in Figure 5 all contribute to the observation of Nogueira et al. [16] that solvent remaining in the gel electrolyte during application has a positive effect on the ionic conductivity and therefore on the efficiency. This leads to the conclusion that drying of the gel electrolyte should be avoided in order to benefit from the increased efficiency due to the remaining solvent until the gel electrolyte is dried incrementally during usage of DSSCs. Even with a strongly reduced amount of solvent, as can be assumed for sample 9, a rudimentary energy conversion efficiency and long-term stability of the DSSC is still provided.

### 3.4. Influence of the PEO-Based Gel Electrolyte on the Long-Term Stability

To evaluate the potential benefit of the gel electrolyte, sample 1 is compared to the reference with a commercial liquid electrolyte. Contrary to the assumption of high ionic conductivity due to the absence of a hindering polymer matrix, the ionic conductivity of the commercial liquid electrolyte of the reference was, with (0.47 ± 0.01) mS/cm (cf. Table 2), surprisingly lower than the ionic conductivity of sample 1 ((2.85 ± 0.01) mS/cm). This could be due to a possibly lower concentration of electrolytic salts, which significantly influences the electrolyte [44], in the unknown composition of the commercial liquid electrolyte.

**Figure 6:**
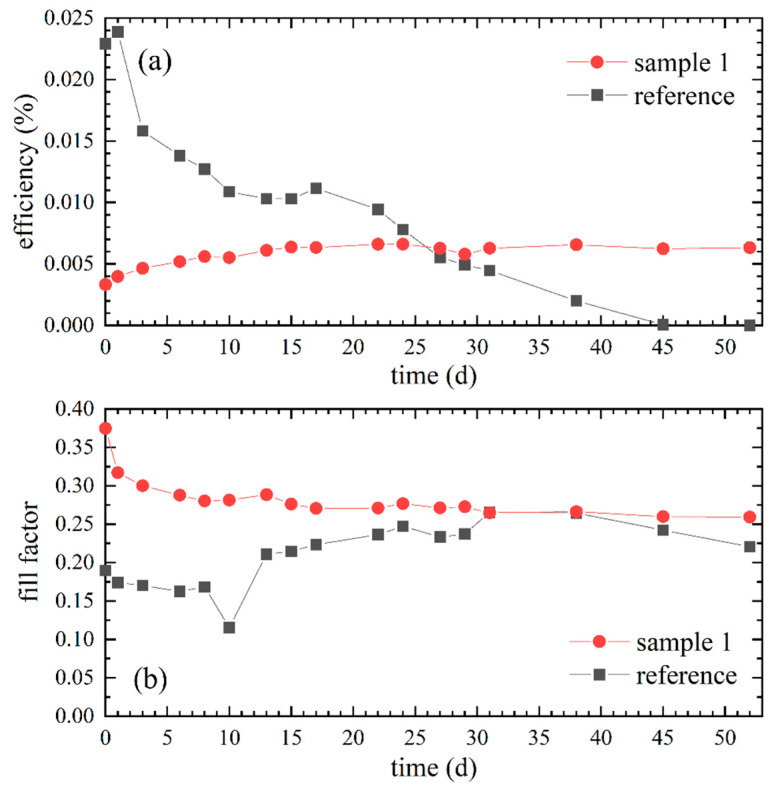
Comparison of sample 1 with 17.4 wt% PEO to the reference with a commercial liquid electrolyte: (**a**) Efficiency values over 52 days; (**b**) Temporal progressions of the related fill factors.

As depicted in Figure 6a, the efficiency of the reference was until day 27 comparatively higher but decreased constantly since day 1. In contrast, the efficiency of sample 1 was relatively low compared to the maximum values of the reference, though it increased slightly during the first 15 days and remained constant thereafter. No decrease in efficiency was measured within the observed 52 days. The fill factor of sample 1 depicted in Figure 6b decreased in the first 17 days, whereas the fill factor of the reference increased approximately until day 22. Afterwards both fill factors remained at relatively similar values.

**Figure 7:**
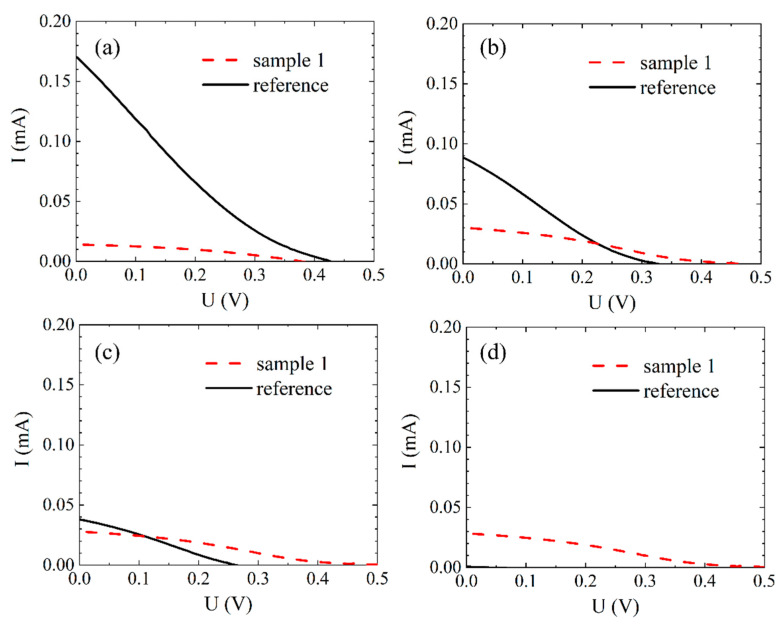
Measured I-U curves of sample 1 and the reference: (**a**) on day 0; (**b**) day 15; (**c**) day 31; (**d**) day 52.

Figure 7 shows the current-voltage measurements of the days 0, 15, 31 and 52. On day 0 (Figure 7a) the short-circuit current (I_SC_) of the reference was about 11 times higher than the current of sample 1. The open-circuit voltages (V_OC_) were relatively similar with approximately 0.42 V for the reference and 0.38 V for sample 1. After 15 days (Figure 7b) the I_SC_ of the reference nearly halved and that of sample 1 almost doubled. Regarding V_OC,_ an increase for sample 1 and a decrease for the reference was observed after 15 days. The increases of I_SC_ and V_OC_ of sample 1 after 15 days are due to the time required for the electrolyte to infiltrate the semiconductor layer completely [21,28,29,47]. In the measurements of the 31st day (Figure 7c), a decreasing trend of I_SC_ and V_OC_ of the reference can be clearly assumed, whereas the values of sample 1 remained relatively constant. The I-U curve of sample 1 on day 52 (Figure 7d) resembles the measurements of day 15 and 31. The respective values of the reference were barely noticeable at this point.

Although the ionic conductivity was significantly lower, the efficiency of the reference was higher in the first 25 days than that of sample 1, which had a higher ionic conductivity. This can be explained by a better penetration of the liquid electrolyte into the porous TiO_2_ layer and thus improved contact between the electrolyte and the dyed semiconductor, without hindering polymers [6,11,16,17,44].

The observed decreases of efficiency, I_SC_ and V_OC_ of the reference DSSC with a commercial liquid electrolyte is caused upon leakage and drying of the electrolyte as it is a common phenomenon of unsealed DSSCs with liquid electrolytes [3,8,10]. Regarding sample 1, no decrease in efficiency, I_SC_ or V_OC_ occurred for 52 days, most plausible due to PEO as gelling agent, which builds an encapsulating framework and therefore prevents leakage as well as limits evaporation [7,8,17,43,48].

With the PEO content in sample 1 a prevented drying for at least 52 days is assumed and with regard to the other PEO-based DSSCs, at least a delay in drying can be supposed. Based on these results, a positive influence of the PEO-based gel electrolyte on the long-term stability of the DSSCs can be clearly conducted. In addition, despite the PEO framework, the fill factor and thus the internal resistance as well as the quality of the DSSC of sample 1 are similar to the reference. Therefore, the gel electrolyte proposed here, even without plasticizers and ionic liquids, which are known to enhance the efficiency, can be seen as a suitable alternative to improve the long-term stability compared to conventional liquid electrolytes [2].

Shi et al. [43] investigated the stability of DSSCs with a slightly lower PEO concentration in the gel electrolyte than in sample 1 over 7 days. They reported a decrease in efficiency of approximately 7.2% after 7 days regarding their DSSCs with a PEO concentration of 15 wt% in the gel electrolyte, which included also ionic liquids. In contrast to this, the efficiency of sample 1 increased slightly after 7 days and no decrease was observed within 52 days. However, it is to mention that Shi et al. recorded an efficiency of 4.18 % whereas the maximum efficiency of sample 1 is roughly 0.007%. This is based on the application of different materials, especially the usage of a natural dye instead of the common highly efficient but toxic dyes.

Furthermore, Xia et al. [48] measured the efficiencies of paraffin wax sealed DSSCs utilizing a gel electrolyte with ionic liquids and a PEO content of 8.6 wt% over 25 days. The efficiencies of those DSSCs decreased over this period by approximately 30%, whereas the efficiency of DSSCs with liquid electrolyte decreased by about 80%. The efficiency of the here built reference DSSC with a commercial liquid electrolyte decreased after 25 days to an extent comparable to Xia et al. And the efficiency of sample 5 (cf. Figure 4a), whose gel electrolyte with a PEO concentration of 8 wt% is comparable to the gel electrolyte of Xia et al., decreased after 25 days by about 23%. This indicates that the DSSCs assembled with our gel electrolyte reach a comparable stability to the DSSCs of Xia et al. despite the omission of sealing and ionic liquids. However, again, it is to mention that the DSSCs used by Xia et al. had efficiencies around 5%, which is much higher than the highest efficiency of about 0.02% presented here, which was achieved with the commercial liquid electrolyte.

The application of a natural dye, namely forest fruit tee, is the main reason for the relatively low efficiencies achieved here. With those natural dyes and non-toxic DSSCs, efficiencies below 0.1% are mostly common [49,50]. This disadvantage of the natural dye can be compensated by its advantageous low costs and environmental friendliness, which enable future, textile-based large-scale applications of otherwise unused surfaces [10,21,22,49,50].

The cost advantage of the natural dye is evident when compared to the ruthenium dye N712, which is one of the few non-hazardous ruthenium dyes and with which 8% efficiency can be reached [51,52]. 50 mg of this dye, which is approximately enough for 10 DSSCs (with 6 cm^2^ each), costs about 148 Euro [53], making it the most expensive component with about 14.8 Euro per DSSC [25]. In contrast, 62.5 g of forest fruit tea were purchased for about 1 Euro, which are sufficient for 75 DSSCs and thus costs as dye per DSSC about 0.013 Euro. Consequently, a cost-effective natural dye, which is by a factor of 10^3^ cheaper per DSSC than N712, enables low-cost and large-scale applications.

In a previous publication it was discovered that the relatively large area of a DSSC, which is here also 6 cm^2^, is another cause for the low efficiency of the DSSCs, besides the absence of a conventional toxic dye [30]. However, if the reference used here is compared with almost identical DSSCs from another previous study, where a maximum efficiency of 0.08% was achieved, it is evident that the DSSCs built here have even lower efficiencies [3]. This is most probably due to a change in the recipe of the purchased forest fruit tea used as dye, which results in lower efficiencies compared to the old product, although the absorption spectra did not differ significantly. Additionally, a different batch of glass electrodes were used, and measurements were performed against a black background, which reduces the measured efficiency compared to a reflective background [54,55]. Furthermore, the known problem of insufficient reproducibility makes it generally difficult to accurately compare DSSCs assembled by different scientists [10,30].

## 4. Conclusions and Outlook

To face the challenges of textile-based DSSCs first experiments with glass electrodes regarding appropriate gel electrolytes are presented here. It is demonstrated that DMSO is a suitable solvent for such a gel electrolyte. Additionally, the results discussed here show that a higher solvent content in the gel electrolyte leads to higher efficiencies but does not prevent solvent evaporation or a decreasing efficiency as much as a higher PEO content. A trend of decreasing ionic conductivity with increasing PEO concentration from 8 wt% to 24 wt% was observed. Among the investigated gel electrolytes, the composition of sample 1 with 17.4 wt% PEO is considered the most suitable. Since this sample showed both a steady long-term stability with a relatively high efficiency and a fill factor similar to a DSSC with a commercial liquid electrolyte. Furthermore, drying the gel electrolyte prior to assembly is considered unsuitable based on the presented results, because remaining solvent results in a positive effect on efficiency. However, even with an intensely dried gel electrolyte (sample 9), the long-term stability of the efficiency is higher in contrast to a commercial liquid electrolyte.

All in all, adequate gel electrolyte compositions with non-toxic and inexpensive components were evaluated, from which appropriate PEO-based gel electrolytes enhancing the long-term stability for environmentally friendly textile-based DSSCs can be concluded. The samples presented here will be measured further in order to examine the stability over a longer time period. In future experiments it is to investigate how the low efficiencies, which are typical for the type of DSSCs presented here, can possibly be further improved by optimizing the gel electrolyte. It must also be researched how a proposed gel electrolyte actually performs in a textile-based DSSC.

## Figures and Tables

**Table 1:** Overview of the samples with the respective compositions of the PEO-based gel electrolytes.

Sample Number	Solvent	Electrolytic Salts (KI + I_2_) (wt%)	PEO (wt%)	Drying
1	DMSO	13.0	17.4	0 h
2	Distilled water	13.0	17.4	0 h
3	DMSO	14.3	9.5	0 h
4	DMSO	12.0	24.0	0 h
5	DMSO	12.0	8.0	0 h
6	DMSO	12.0	8.0	0.5 h
7	DMSO	12.0	8.0	1 h
8	DMSO	12.0	8.0	2 h
9	DMSO	13.0	17.4	2 h, 80 °C, oven
reference	-	Man Solar electrolyte	-	-

**Table 2:** Ionic conductivities of the gel electrolyte solutions and the liquid Man Solar electrolyte of the reference measured at 40 °C.

Sample Number	Ionic Conductivity (mS/cm)
1	2.85 ± 0.01
2	4.76 ± 0.01
3	3.15 ± 0.01
4	2.71 ± 0.01
5	3.21 ± 0.01
reference	0.47 ± 0.01
